# Linking the timing of a mother’s and child’s death: Comparative evidence from two rural South African population-based surveillance studies, 2000–2015

**DOI:** 10.1371/journal.pone.0246671

**Published:** 2021-02-08

**Authors:** Brian Houle, Chodziwadziwa W. Kabudula, Alan Stein, Dickman Gareta, Kobus Herbst, Samuel J. Clark

**Affiliations:** 1 School of Demography, The Australian National University, Canberra, Australia; 2 Faculty of Health Sciences, MRC/Wits Rural Public Health and Health Transitions Research Unit (Agincourt), School of Public Health, University of the Witwatersrand, Johannesburg, South Africa; 3 CU Population Center, Institute of Behavioral Science, University of Colorado at Boulder, Boulder, Colorado, United States of America; 4 Section of Child and Adolescent Psychiatry, Department of Psychiatry, University of Oxford, Oxford, United Kingdom; 5 Africa Health Research Institute, KwaZulu-Natal, South Africa; 6 Department of Sociology, The Ohio State University, Columbus, Ohio, United States of America; University of Botswana, BOTSWANA

## Abstract

**Background:**

The effect of the period before a mother’s death on child survival has been assessed in only a few studies. We conducted a comparative investigation of the effect of the timing of a mother’s death on child survival up to age five years in rural South Africa.

**Methods:**

We used discrete time survival analysis on data from two HIV-endemic population surveillance sites (2000–2015) to estimate a child’s risk of dying before and after their mother’s death. We tested if this relationship varied between sites and by availability of antiretroviral therapy (ART). We assessed if related adults in the household altered the effect of a mother’s death on child survival.

**Findings:**

3,618 children died from 2000–2015. The probability of a child dying began to increase in the 7–11 months prior to the mother’s death and increased markedly in the 3 months before (2000–2003 relative risk = 22.2, 95% CI = 14.2–34.6) and 3 months following her death (2000–2003 RR = 20.1; CI = 10.3–39.4). This increased risk pattern was evident at both sites. The pattern attenuated with ART availability but remained even with availability at both sites. The father and maternal grandmother in the household lowered children’s mortality risk independent of the association between timing of mother and child mortality.

**Conclusions:**

The persistence of elevated mortality risk both before and after the mother’s death for children of different ages suggests that absence of maternal care and abrupt breastfeeding cessation might be crucial risk factors. Formative research is needed to understand the circumstances for children when a mother is very ill or dies, and behavioral and other risk factors that increase both the mother and child’s risk of dying. Identifying families when a mother is very ill and implementing training and support strategies for other members of the household are urgently needed to reduce preventable child mortality.

## Background

Improving maternal and child survival remains an urgent global priority. In 2017 over five million children younger than five years of age died globally, with about 50% of under-five deaths occurring in sub-Saharan Africa [1]. In the sub-Saharan African region women also face high levels of mortality, particularly due to HIV/AIDS and maternal-related conditions [2]. This is compounded by projections that the child population in sub-Saharan Africa is expected to increase rapidly in the next 20 years, which may increase the number of under-five deaths [1].

The death of a mother has a major impact on the health and survival of her children. Most prior research examining the timing of the death of a mother and her child has concentrated on the period following the mother’s death [3–7]. Emerging evidence also suggests critical risk periods for children before the mother dies. An analysis of pooled data from three HIV cohort studies in East Africa found that when a mother died, mortality risks tripled for her children in the two years surrounding her death [8]. However, the study was unable to isolate an effect for the period prior to the mother’s death. Another study examining time periods before the HIV epidemic and during its peak found that children were at high risk of dying not only after their mother’s death but also in the months prior when she was seriously ill, peaking at the month of her death [9]. However, as that study predated the widespread availability of antiretroviral therapy (ART) in the study setting, it is unclear whether and if so how ART availability changes the relationship between the timing of a mother’s death and child mortality in HIV-endemic areas.

The extent to which studies are generalizable across settings is also unclear [10,11], particularly given differences in household organization, available resources and care-taking practices. This was highlighted in a recent meta-analysis of child mortality risk following a mother’s death [12]. The authors found unexplained variation between studies that may be due to context-specific factors, as well as variation in study designs, methodologies, and reporting of results that limited their ability to pool estimates. There are also limited comparisons within countries and disadvantaged populations [13]. Understanding if and how the temporal relationship between maternal and child death varies between settings carries important implications for policies and interventions to reduce preventable child mortality. Obviously, efforts to treat the mother herself would be a high priority. However, if evidence indicates that the period of illness before a mother’s death puts her young children at high risk of dying, programs need to identify mothers and their families and intervene when a mother becomes very ill and is unable to care and feed her children, in addition to providing support for the children after the mother dies. Comparative evidence is critical to inform such interventions.

We use prospective data over a 16-year period from two demographic surveillance sites (DSS) in rural South Africa with high levels of HIV prevalence. Critically, the two sites differed in key aspects as to when ART became widely available. This allows us to assess the comparability of the temporal patterns between mother and child death, as well as to examine the impact of ART on potentially changing these associations. We also assess if the presence of related adults who may play a substitution caregiving role attenuates the temporal pattern along with household socio-economic status.

## Methods

### Setting and data sources

We used data from two DSSs in rural South Africa: (1) the Agincourt Health and socio-Demographic Surveillance System [14] (AHDSS) in Agincourt sub-district in rural Mpumalanga Province and (2) the Africa Health Research Institute [15] (AHRI) in uMkanyakude in rural KwaZulu-Natal Province. Each DSS monitors a geographically defined population over time and collects prospective, longitudinal demographic data, including fertility, mortality, and migration, along with social indicators and household-level information. For both AHDSS and AHRI participation rates have remained virtually complete (>99%).

Population surveillance in AHDSS began in 1992 –in 2011 the population under surveillance was approximately 90,000 people residing in 27 villages. Updates were conducted at intervals of about 15–18 months before 1999, and annually since 1999. The surveillance area is located in the Ehlanseni district of Mpumalanga and the dominant ethnic group are the amaShangaan. While infrastructure in the area is limited and unemployment is high, there have been steady increases in socio-economic status and access to electricity over time [16]. HIV prevalence is high in the population (19.4% in 2011) [17], and until recently mortality in the site was increasing in children and young and middle-aged adults [18]. Clinics within the study area began providing ART in 2007. Further details on AHDSS data collection and quality control procedures are available in Kahn et al [14].

AHRI began population surveillance in 2000 –with a surveillance population of approximately 90,000 people in 2011. Updates were conducted two times a year until 2012, and three times a year since 2012. The surveillance area is located in the Umkanyakude district of KwaZulu-Natal and the population is almost entirely Zulu-speaking. The main sources of income are waged employment and state pensions. The area has experienced significant increases in the availability of electricity and toilet facilities over time. HIV prevalence is high in the population: estimated at 29% (ages 15–49) in 2011 [19]. ART first became widely available in the study area in 2004 –which significantly increased life expectancy in the population [20]. Further details on AHRI data collection and quality control procedures are available in Tanser et al [15] and elsewhere [21].

In both DSSs, a verbal autopsy (VA) was conducted for individuals who died between surveillance rounds. Using a standardized VA instrument, a specially trained fieldworker or nurse conducted a VA interview with the closest living relative to record signs and symptoms experienced by the decedent before their death.

Data for household socioeconomic status (SES) comes from a common set of household indicators from each DSS measured since 2001. For AHDSS, SES was measured every two years from 2001, and annually since 2013. For AHRI, SES was measured annually, except for 2000, 2002, 2004, and 2008. Links between mother and child are direct for both sites through pregnancy outcome forms. By linking records through the mother’s identification number, we have data describing the presence of maternally-related family members in the household.

Ethics approval for AHDSS was obtained from the Human Research Ethics Committee (Medical) of the University of the Witwatersrand, Johannesburg, South Africa (protocols M960720 and M110138). Ethics approval for AHRI was obtained from the Biomedical Research Ethics Committee (BREC) of the University of KwaZulu-Natal, Durban, South Africa (reference number BE169/15).

For both AHDSS and AHRI, informed verbal consent is obtained at each surveillance visit from the head of the household or proxy adult respondent. This is recorded in the data collection instruments. The verbal consent process is standard across all INDEPTH (www.indepth-network.org) DSSs, given the impossibility of contacting every person in the DSS. The verbal consenting process has continued to be accepted by the aforementioned ethics committees.

### Statistical analysis

Our analysis covered the time period 2000–2015. We modelled a child’s risk of dying before the age of five years using discrete time event history analysis—where each child was at risk of dying for each month they were observed [22]. We organised the data as child-months for children under five years of age, including one observation for each observed child-month. While uncommon at both sites [23–25], children who migrated out of the study sites were considered right censored, or interval censored if they returned at a later date. Our covariates included time-constant and time-varying factors defined at the beginning of each child month. We modelled the monthly probability of a child dying using multi-level [26] relative risk regression [27] with random mother intercepts to account for shared mortality risks of children with the same mother. We included time constant covariates of child sex, multiple birth, mother’s age at birth, and an indicator of DSS; and time varying covariates of child age, time period, timing of mother’s death, number of other under-five children in the household, and the presence or absence of father, older brother or sister, and maternal aunt, uncle, or grandmother. The data were split into three time periods: 2000–2003, 2004–2007, and 2008–2015. The split between 2003 and 2004 marks the beginning of ART availability at AHRI, while the split between 2007 and 2008 marks the beginning of ART availability at AHDSS. This permits an ecological examination of the impact of ART availability on the observed temporal associations. For the timing of mother’s death, we categorized periods before and after her death, with the referent being mother alive or will die 12 or more months in the future. We tested for interactions between timing of mother’s death and DSS, time period, and child age group and sex using nested likelihood ratio tests. For the child age interaction, we collapsed the <1 and 1 to 5-month age groups together and recategorized timing of mother’s death to 0, 1–2, 3–5, and 6+ months for periods before and after her death to ensure adequate representation in each cell. To allow for the risk of dying to change over time differently between the sites, we also tested for an interaction between DSS and time period.

To assign causes of death, we used InterVA-5 [28]–a model that identifies up to three causes of death that are consistent with the observed signs and symptoms in a more standardised and automated manner compared to physician-coded cause of death. We used the single cause with the largest likelihood for each death with a complete VA interview. We excluded accidental maternal deaths and stillbirths given differences in the causes and context compared to other causes. Child cause of death distributions were compared using Fisher’s exact test. Mother cause of death distributions were categorised according to South African burden of disease classification system [29].

We also estimated a secondary model including household SES as a time-varying covariate, based on a household wealth index, to determine if SES variation explained our main finding. We computed the household wealth index by summarizing the common set of household asset indicators between the two sites using principal components analysis [16]. For each DSS and year (2001–2015), we summarized household SES using tertiles of the first principal component score based on the most recent measurement. We conducted all analyses using Stata 15 [30].

## Results

**Fig 1:**
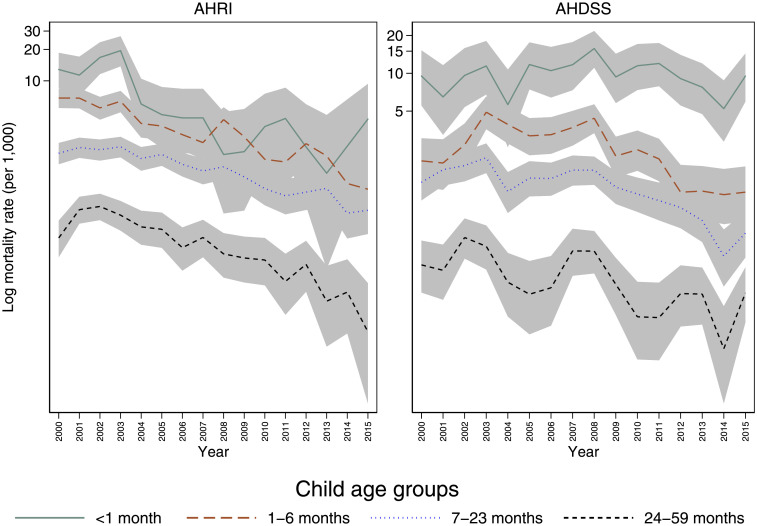
Child mortality rate (per 1,000) by year and child age for the Agincourt Health and Socio-Demographic Surveillance System (AHDSS) and the Africa Health Research Institute (AHRI), South Africa 2000–2015.

Table 1 presents sociodemographic characteristics of all the children included in the study from both DSSs. A total of 3,618 children died between 2000 and 2015. Mothers of 6,010 children died in the first five years of their child’s life (6%). Fig 1 shows that child mortality began declining at AHRI in 2004 and AHDSS in 2008.

**Table 1:** Characteristics of children and mothers by maternal survival, Agincourt Health and Socio-Demographic Surveillance System (AHDSS) and the Africa Health Research Institute (AHRI), South Africa 2000–2015.

	Mother survived		Mother died		Total	
	N	(%)	N	(%)	N	(%)
**Children**	95,101		6,010		101,111	
Child death	2,897		721		3,618	
Child age at death (months)						
<1	448	(15.5)	64	(8.9)	512	(14.2)
1–6	854	(29.5)	273	(37.9)	1,127	(31.1)
7–23	1,155	(39.9)	254	(35.2)	1,409	(38.9)
24–59	440	(15.2)	130	(18.0)	570	(15.8)
DSS						
AHRI	42,485	(44.7)	4,180	(69.6)	46,665	(46.2)
AHDSS	52,616	(55.3)	1,830	(30.4)	54,446	(53.8)
Sex of child						
Girl	47,382	(49.8)	3,039	(50.6)	50,421	(49.9)
Boy	47,719	(50.2)	2,971	(49.4)	50,690	(50.1)
Multiple birth						
Singleton	91,841	(96.6)	5,760	(95.8)	97,601	(96.5)
Multiple	3,260	(3.4)	250	(4.2)	3,510	(3.5)
Number of other household children^a^						
0	37,702	(39.6)	2,220	(36.9)	39,922	(39.5)
1	32,546	(34.2)	1,961	(32.6)	34,507	(34.1)
2	14,763	(15.5)	1,034	(17.2)	15,797	(15.6)
3+	10,090	(10.6)	795	(13.2)	10,885	(10.8)
Household SES^a^						
Low	12,504	(32.5)	568	(36.7)	13,072	(32.7)
Middle	12,771	(33.2)	501	(32.4)	13,272	(33.2)
High	13,164	(34.2)	479	(30.9)	13,643	(34.1)
**Mothers**	57,803		3,851		61,654	
DSS						
AHRI	23,586	(40.8)	2,687	(69.8)	26,273	(42.6)
AHDSS	34,217	(59.2)	1,164	(30.2)	35,381	(57.4)
Mother’s age at birth (years)						
15–19	19,360	(33.5)	837	(21.7)	20,197	(32.8)
20–24	17,915	(31.0)	1,084	(28.1)	18,999	(30.8)
25–29	10,017	(17.3)	906	(23.5)	10,923	(17.7)
30–34	5,921	(10.2)	562	(14.6)	6,483	(10.5)
35+	4,590	(7.9)	462	(12.0)	5,052	(8.2)

^a^ Based on one unique record per child, variable is time-varying.

The results from the full model are presented in Table 2. An interaction between DSS and months before/after mother’s death (p<0.001), time period and months before/after mother’s death (p<0.001), and DSS and time period significantly improved model fit (p<0.001). A multi-level model including a mother random intercept improved model fit according to the Bayesian Information Criterion (Δ*BIC* = 139) and resulted in the final model. Interactions between child age or gender and months before/after mother’s death did not significantly improve model fit. Boys have a 12% higher risk of dying compared to girls (95% confidence interval [1.1, 1.2]). Younger children, being part of a multiple birth, and being born to an older mother increased the risk of dying. Children in households with higher numbers of other children also have an increased mortality risk.

**Table 2:** Multilevel relative risk regression of child death on timing of mother death and controls, Agincourt Health and Socio-Demographic Surveillance System (AHDSS) and the Africa Health Research Institute (AHRI), South Africa 2000–2015 (n = 3,687,035 child months).

Variable	RRR	95% CI	p-value
Months before/after mother’s death			
Alive or 12+ mo before	1.000	-	-
7 to 11 mo before	4.197	[2.543, 6.928]	<0.001
4 to 6 mo before	9.422	[5.753, 15.432]	<0.001
1 to 3 mo before	14.782	[9.537, 22.912]	<0.001
Month of mother’s death	29.962	[16.652, 53.910]	<0.001
1 to 3 mo after	10.121	[5.387, 19.015]	<0.001
4 to 6 mo after	9.824	[4.555, 21.187]	<0.001
7+ mo after	6.475	[3.434, 12.212]	<0.001
Site			
AHRI	1.000	-	-
AHDSS	0.734	[0.648, 0.831]	<0.001
Interactions between months before/after mother’s death and site			
7 to 11 mo before x AHDSS	1.288	[0.621, 2.671]	0.497
4 to 6 mo before x AHDSS	1.241	[0.562, 2.739]	0.594
1 to 3 mo before x AHDSS	2.254	[1.177, 4.316]	0.014
Month of mother’s death x AHDSS	3.954	[1.666, 9.383]	0.002
1 to 3 mo after x AHDSS	3.953	[1.515, 10.317]	0.005
4 to 6 mo after x AHDSS	4.596	[1.416, 14.918]	0.011
7+ mo after x AHDSS	1.296	[0.513, 3.272]	0.583
Time period			
2000 to 2003	1.000	-	-
2004 to 2007	0.593	[0.528, 0.667]	<0.001
2008 to 2015	0.339	[0.302, 0.380]	<0.001
Interactions between months before/after mother’s death and time period			
7 to 11 mo before x 2004 to 2007	1.574	[0.789, 3.144]	0.198
4 to 6 mo before x 2004 to 2007	1.058	[0.508, 2.205]	0.879
1 to 3 mo before x 2004 to 2007	0.678	[0.349, 1.316]	0.251
Month of mother’s death x 2004 to 2007	0.558	[0.230, 1.355]	0.198
1 to 3 mo after x 2004 to 2007	0.703	[0.277, 1.782]	0.457
4 to 6 mo after x 2004 to 2007	0.543	[0.168, 1.758]	0.308
7+ mo after x 2004 to 2007	0.85	[0.368, 1.965]	0.705
7 to 11 mo before x 2008 to 2015	0.656	[0.253, 1.702]	0.386
4 to 6 mo before x 2008 to 2015	0.401	[0.145, 1.111]	0.079
1 to 3 mo before x 2008 to 2015	0.244	[0.100, 0.591]	0.002
Month of mother’s death x 2008 to 2015	0.157	[0.050, 0.491]	0.001
1 to 3 mo after x 2008 to 2015	0.157	[0.043, 0.573]	0.005
4 to 6 mo after x 2008 to 2015	0.13	[0.029, 0.596]	0.009
7+ mo after x 2008 to 2015	0.42	[0.168, 1.054]	0.065
Interactions between site and time period			
AHDSS x 2004 to 2007	1.546	[1.293, 1.848]	<0.001
AHDSS x 2008 to 2015	1.744	[1.476, 2.059]	<0.001
Sex of child			
Girl	1.000	-	-
Boy	1.122	[1.048, 1.202]	0.001
Child age (months)			
<1	1.000	-	-
1–6	0.378	[0.340, 0.420]	<0.001
7–23	0.176	[0.158, 0.195]	<0.001
24–59	0.033	[0.029, 0.038]	<0.001
Multiple birth			
Singleton	1.000	-	-
Multiple	1.822	[1.562, 2.125]	<0.001
Mother’s age at birth (years)			
15–19	1.000	-	-
20–24	1.124	[1.016, 1.243]	0.024
25–29	1.384	[1.234, 1.552]	<0.001
30–34	1.218	[1.058, 1.403]	0.006
35+	1.405	[1.202, 1.643]	<0.001
Number of other children in household			
0	1.000	-	-
1	1.096	[1.010, 1.189]	0.027
2	1.171	[1.057, 1.298]	0.003
3+	1.34	[1.191, 1.507]	<0.001
Father presence			
Absent	1.000	-	-
Present	0.527	[0.479, 0.581]	<0.001
Older brother presence			
Absent	1.000	-	-
Present	0.923	[0.843, 1.010]	0.082
Older sister presence			
Absent	1.000	-	-
Present	0.991	[0.906, 1.085]	0.85
Maternal grandmother presence			
Absent	1.000	-	-
Present	0.859	[0.774, 0.953]	0.004
Maternal uncle presence			
Absent	1.000	-	-
Present	0.923	[0.836, 1.020]	0.115
Maternal aunt presence			
Absent	1.000	-	-
Present	0.994	[0.899, 1.098]	0.905
	Parameter		
$\sigma_{mother}^{2}$	1.009	[0.840, 1.211]	-

**Fig 2:**
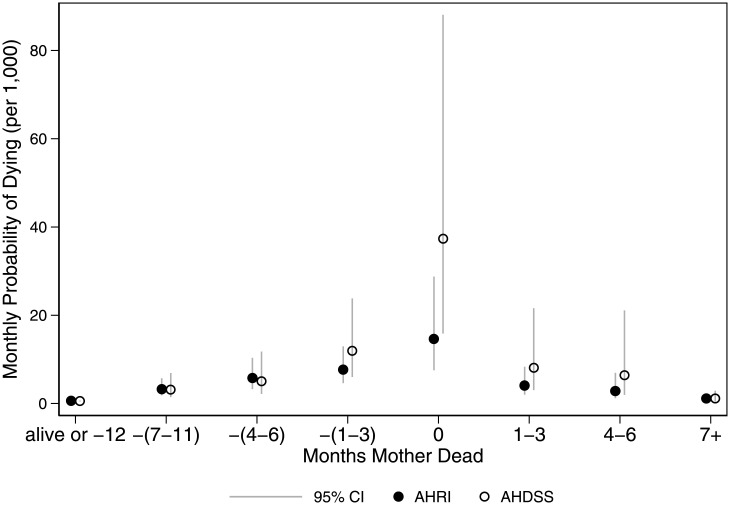
Monthly probability of child death (2000–2015), by months before/after mother’s death and demographic surveillance site: Agincourt Health and Socio-Demographic Surveillance System (AHDSS) and Africa Health Research Institute (AHRI), South Africa 2000–2015. Jittered points to reduce over plotting.

Fig 2 shows model-based predicted probabilities of a child dying by timing of a mother’s death and DSS. The probability of a child dying begins to increase in the 7–11 months prior to the mother’s death, peaks in the month of her death, and remains high for the 1–3 months after her death. Comparisons between the two sites shows that this temporal pattern is evident at both sites, but with a slightly elevated effect at AHDSS compared to AHRI.

**Fig 3:**
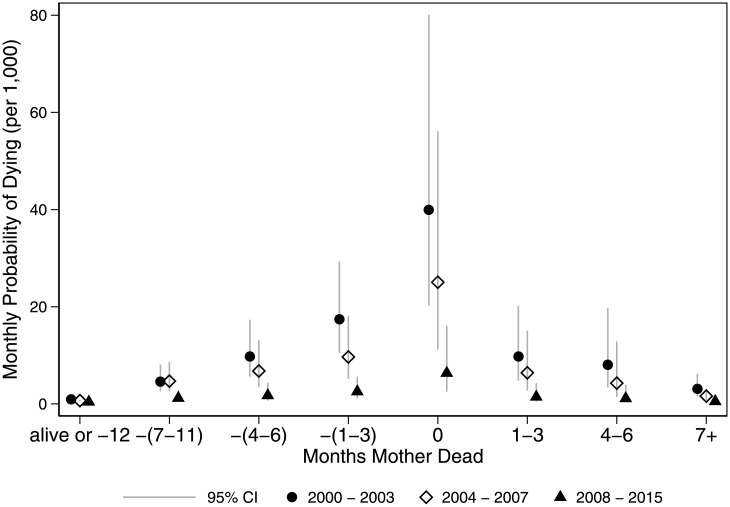
Monthly probability of child death (2000–2015), by months before/after mother’s death and time period. Jittered points to reduce over plotting.

Fig 3 shows predicted probabilities of a child dying by timing of a mother’s death and time period. The probability of dying associated with the temporal pattern of a mother’s death attenuates over time—decreasing in 2004–2007 (when ART became available at AHRI) and 2008–2015 (when ART was available at both sites) relative to 2001–2003. However, the pattern of increased risk before the mother’s death, with elevated risks in the 6-month window around her death still persists in the latest time period (e.g., comparing marginal means in 2008–2015: -(1–3) RRR = 5.4 [2.6, 11.3]; 0 RRR = 9.3 [3.8, 23.0]; 1–3 RRR = 3.2 [1.1, 8.9]; relative to children with mothers who survive or will die 12+ months in the future). Full comparisons by time period are provided in S1 Fig.

Table 2 shows that the presence of either the father (RRR = 0.5 [0.5, 0.6]) or maternal grandmother (RRR = 0.9 [0.8, 0.9]) in the household reduced a child’s risk of dying. However, their presence did not attenuate the temporal associations between a mother’s death and child mortality. The presence of other related household members did not influence child mortality risk.

Finally, in a sub-model including household SES (see S1 Table)–children in the wealthiest tertile have a 22% lower risk of dying compared to children in the poorest tertile (CI [0.70, 0.9]). Household SES is independent of the temporal associations between a mother’s death and child mortality.

Table 3 shows causes of child deaths according to their mother’s survival status. Children whose mothers died over 3 months previously most commonly died due to HIV/AIDS and TB and nutritional disorders. Causes of mother deaths are shown in S2 Table.

**Table 3:** Child causes of death according to months before/after mother’s death. Child causes of death classified by InterVA-5 based on VA.

	Alive or 12+ mos before		11 to 3 mos before		2 mos before to 2 mos after		3+ mos after		
	N	(%)	N	(%)	N	(%)	N	(%)	p-value
HIV/AIDS and TB	824	(25)	31	(31)	21	(22)	26	(45)	0.003
Diarrhoeal diseases	215	(6)	5	(5)	4	(4)	0	(0)	0.158
Respiratory infections	979	(29)	29	(29)	25	(26)	12	(21)	0.542
Other infectious diseases	317	(9)	5	(5)	2	(2)	3	(5)	0.019
Nutritional disorders	61	(2)	4	(4)	1	(1)	4	(7)	0.024
Perinatal period disorders^a^	223	(7)	6	(6)	11	(12)	1	(2)	0.125
External	85	(3)	0	(0)	3	(3)	1	(2)	0.374
Other	77	(2)	0	(0)	0	(0)	1	(2)	0.230
Unknown	584	(17)	20	(20)	28	(30)	10	(17)	0.029

^a^ Includes conditions such as premature, birth asphyxia, and congenital malformation.

## Discussion

Our findings show evidence that children are equally vulnerable in the period before a mother’s death as afterwards. The temporal pattern was evident in two different rural South African settings using harmonized data and a unified statistical framework, with variation in the strength of the relationship. The availability of ART attenuated this pattern over time, although the pattern remained despite widespread availability of ART at both sites. While the presence of the father, maternal grandmother, and being in the highest SES households protected vulnerable children, they were independent of the relationship between the timing of mother and child death. Our findings of increased mortality risk for boys, younger children, and lower SES households align with other published studies from the two sites [31,32].

A likely major underlying cause for the temporal relationship between mother and child death is abrupt cessation of breastfeeding [33], especially for very young children. However, as the effect of timing did not vary by child age, a number of other factors likely contribute to excess mortality risk. Mothers who are ill may have not only stopped breastfeeding, but also been unable to arrange alternative feeding or care [10]. The fact that increased risk began in the year before the mother’s death and remained very high in the few months either side of her death suggests that other potential caregivers may lack the awareness, support, or resources to adequately care for vulnerable children.

Time periods associated with ART rollout across the two sites attenuated the relationship between mother and child death, though the pattern remained even with widespread ART availability at both sites. With the success of prevention of mother-to-child transmission programs, the lowered probability of dying as ART became available suggests that ART was critical to reducing deaths of mothers and their children. Similarly, studies on maternal mortality trends at both sites have shown declines over time—though the rates remain above international targets [34,35]. Two studies from AHRI on the population and individual-level impacts of maternal ART showed the direct benefits of ART on improving child survival [36,37]. A pooled analysis of 21 studies of 24-month child mortality also showed the importance of maternal ART, breastfeeding, and maternal survival on child mortality [38]. Their finding that maternal vital status remained an important independent predictor of child mortality is in-line with our finding that the temporal relationship remained even in the latest time periods with widespread ART availability.

Similar to other studies, we show that the presence of some (maternal grandmothers and fathers) but not all kin are protective for young children [39]. The protective effect of fathers is particularly salient given the high rates of father absence in South Africa. However, as highlighted by other studies, our indicator of father presence over-simplifies the role and support provided by fathers in general and particularly those non-coresident in the household [40–43]. However, the presence of either the maternal grandmother or father did not alter the effect of the timing of the mother’s death on a child’s risk of dying. This is likely due to more proximate factors around the time of a mother’s death, including cessation of breastfeeding and diverted resources and attention towards the mother when she is very ill.

Education, training, and support for families and the local community around care for children when the mother is ill represents a key opportunity for intervention. If well supported by training and supervision [44,45], community health workers may play a critical role in the identification and support of high-risk families where a mother is unwell. Identification and support of at-risk families was also recommended by local panels of healthcare and community representatives from a study in Mali and Uganda [46]. Palliative care programs that are integrated with community-based health and social services could also be an avenue to provide support to families on child nutrition and care [47–49].

While we show comparative evidence from two rural South African settings, further studies using an expanded number of settings and countries are needed to assess wider generalizability. This is particularly important given the likely importance of different contextual factors that may influence critical risk periods for children around a mother’s death, as well as the role of context in successful intervention implementation [50].

We acknowledge study limitations. We did not have information on child nutrition and caretaking, which limits our ability to understand the exact mechanisms linking maternal illness and death to child mortality. Studies are urgently needed to understand the factors that put both mothers and their children at risk, and family responses when a mother is very ill or dies [5]. We were also not able to include HIV status of individuals as routine HIV testing is not done at AHDSS. However, a study from AHRI on the impact on child mortality restricted to after a mother’s death found an independent association net of maternal HIV status [32]. This also aligns with earlier evidence from Tanzania of an independent effect of a mother’s death regardless of HIV status [51]. Differences in the frequency of data collection between the two sites (one round per year in AHDSS versus two-to-three rounds per year in AHRI) may also have affected our estimates. For instance, some neonatal and infant births may not be recorded if the birth and death occur between consecutive household visits. Evidence comparing data from an HDSS linked with antenatal care registers found that while missing pregnancy status and outcomes will likely be captured in future rounds if resulting in a live birth, if they resulted in a child death they may be not captured by the HDSS [52]. While both AHDSS and AHRI have careful quality control procedures in place, including pre-populated household rosters and careful probing about the pregnancy status of every woman of childbearing age, our estimates for very young children are likely conservative. Further, as both sites use proxy respondents when updating household rosters and vital events, this may limit the accuracy of reported dates of birth and death. While our use of InterVA to assign causes of death facilitates temporal consistency, there may be limitations in its ability to assign causes of death. However, a study from AHDSS comparing InterVA with physician-coded causes of death showed broad alignment between the two approaches [53]. Our causes of child death also broadly align with estimates from the South African Global Burden of Disease study [54]. The timing of a mother’s death and child survival may be due to the shared environment or other common risk factors. While our models adjusted for household SES showed similar results, some residual confounding may remain. We also used multi-level modelling to account for clustering of household child mortality risk, as studies have highlighted the importance of unobserved heterogeneity in examining child mortality risks [55,56]. We did not examine periods of severe maternal illness that did not result in a mother’s death as we lack comparative information on disability and morbidity. This means our child mortality estimates when the mother is severely ill are likely conservative.

## Conclusions

The six-month window surrounding a mother’s death is a period of substantial risk for children’s survival. The availability of ART and the resulting survival gains for mothers and their children substantially reduced this effect, but there remains an elevated period of mortality risk, particularly for young children. While the presence of other family members can support children, evidence from this study suggests that proactive, community-based interventions are urgently needed to support these families to protect vulnerable children both when a mother becomes very ill and in the immediate period following her death.

## Supporting information

S1 Fig Marginal mean relative mortality risk ratios (2000–2015), by months before/after mother’s death and time period. Referent marked by dotted line for children whose mother survives or will die 12+ months in the future. Scale of the horizontal axis varies between each time period.
(PDF)

S1 Table Multilevel relative risk regression of child death on timing of mother death and controls, Agincourt Health and Socio-Demographic Surveillance System (AHDSS) and Africa Health Research Institute (AHRI), South Africa 2001–2015 (n = 2,655,610 child months). Estimation sample for each model restricted to those with household SES information.
(DOCX)

S2 Table Mother causes of death, classified by InterVA-5 based on VA. Causes of death categorised according to the South African burden of disease classification system.
(DOCX)
