# Supplementary material for: Linking the timing of a mother’s and child’s death: Comparative evidence from two rural South African population-based surveillance studies, 2000–2015
Source: PLoS One. 2021 Feb 8;16(2):e0246671. doi: 10.1371/journal.pone.0246671 (PMC7869981; doi:10.1371/journal.pone.0246671)

S1 Figure. Marginal mean relative mortality risk ratios (2000-2015), by months before/after mother's death and time period. Referent marked by dotted line for children whose mother survives or will die 12+ months in the future. Scale of the horizontal axis varies between each time period.

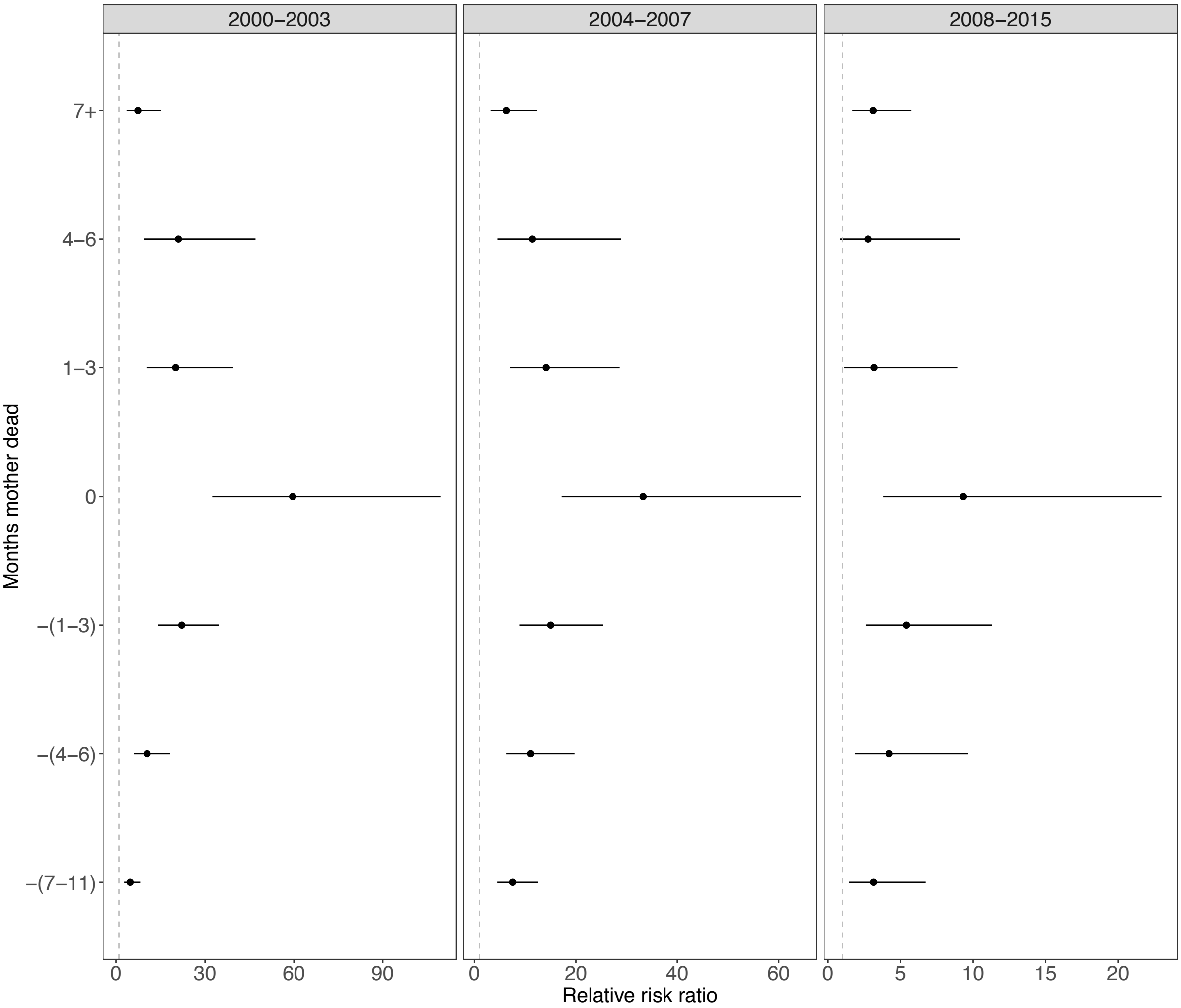

Supplement: S1 Fig — Referent marked by dotted line for children whose mother survives or will die 12+ months in the future. Scale of the horizontal axis varies between each time period. (PDF) [file pone.0246671.s001.pdf]
