# Supplementary material for: Linking the timing of a mother’s and child’s death: Comparative evidence from two rural South African population-based surveillance studies, 2000–2015
Source: PLoS One. 2021 Feb 8;16(2):e0246671. doi: 10.1371/journal.pone.0246671 (PMC7869981; doi:10.1371/journal.pone.0246671)
Supplement: S1 Table — Estimation sample for each model restricted to those with household SES information. (DOCX) [file pone.0246671.s002.docx]

**S1 Table. Multilevel relative risk regression of child death on timing of mother death and controls, Agincourt Health and Socio-Demographic Surveillance System (AHDSS) and Africa Health Research Institute (AHRI), South Africa 2001-2015 (n=2,655,610 child months).** Estimation sample for each model restricted to those with household SES information.

|  | **Without SES** | | | **With SES** | | |
| --- | --- | --- | --- | --- | --- | --- |
| **Variable** | **RRR** | **95% CI** | **p-value** | **RRR** | **95% CI** | **p-value** |
| Months before/after mother’s death |  |  |  |  |  |  |
| Alive or 12+ mo before | 1.000 | - | - | 1.000 | - | - |
| 7 to 11 mo before | 4.047 | [1.989, 8.235] | <0.001 | 4.02 | [1.976, 8.176] | <0.001 |
| 4 to 6 mo before | 11.827 | [6.554, 21.343] | <0.001 | 11.808 | [6.545, 21.302] | <0.001 |
| 1 to 3 mo before | 12.625 | [6.799, 23.442] | <0.001 | 12.63 | [6.808, 23.432] | <0.001 |
| Month of mother’s death | 27.13 | [11.902, 61.839] | <0.001 | 26.915 | [11.803, 61.373] | <0.001 |
| 1 to 3 mo after | 4.605 | [1.538, 13.786] | 0.006 | 4.582 | [1.531, 13.710] | 0.006 |
| 4 to 6 mo after | 9.831 | [3.968, 24.358] | <0.001 | 9.848 | [3.978, 24.379] | <0.001 |
| 7+ mo after | 5.427 | [2.669, 11.034] | <0.001 | 5.421 | [2.666, 11.023] | <0.001 |
| Site |  |  |  |  |  |  |
| AHRI | 1.000 | - | - | 1.000 | - | - |
| AHDSS | 0.761 | [0.649, 0.892] | 0.001 | 0.772 | [0.658, 0.906] | 0.002 |
| Interactions between months before/after mother’s death and site | 1.000 | - | - | 1.000 | - | - |
| 7 to 11 mo before x AHDSS | 0.878 | [0.342, 2.252] | 0.786 | 0.841 | [0.328, 2.159] | 0.719 |
| 4 to 6 mo before x AHDSS | 1.326 | [0.532, 3.308] | 0.545 | 1.282 | [0.514, 3.196] | 0.595 |
| 1 to 3 mo before x AHDSS | 2.089 | [0.916, 4.762] | 0.08 | 2.034 | [0.893, 4.632] | 0.091 |
| Month of mother’s death x AHDSS | 6.578 | [2.011, 21.517] | 0.002 | 6.462 | [1.978, 21.111] | 0.002 |
| 1 to 3 mo after x AHDSS | 7.559 | [2.148, 26.593] | 0.002 | 7.37 | [2.093, 25.953] | 0.002 |
| 4 to 6 mo after x AHDSS | 4.787 | [1.244, 18.416] | 0.023 | 4.687 | [1.219, 18.019] | 0.025 |
| 7+ mo after x AHDSS | 1.308 | [0.455, 3.756] | 0.618 | 1.279 | [0.446, 3.669] | 0.647 |
| Time period |  |  |  |  |  |  |
| 2000 to 2003 | 1.000 | - | - | 1.000 | - | - |
| 2004 to 2007 | 0.572 | [0.490, 0.668] | <0.001 | 0.57 | [0.488, 0.666] | <0.001 |
| 2008 to 2015 | 0.292 | [0.248, 0.344] | <0.001 | 0.291 | [0.247, 0.342] | <0.001 |
| Interactions between months before/after mother’s death and time period |  |  |  |  |  |  |
| 7 to 11 mo before x 2004 to 2007 | 1.574 | [0.601, 4.121] | 0.356 | 1.582 | [0.604, 4.145] | 0.351 |
| 4 to 6 mo before x 2004 to 2007 | 0.73 | [0.286, 1.862] | 0.51 | 0.721 | [0.283, 1.840] | 0.494 |
| 1 to 3 mo before x 2004 to 2007 | 0.681 | [0.280, 1.656] | 0.397 | 0.668 | [0.275, 1.624] | 0.374 |
| Month of mother’s death x 2004 to 2007 | 0.344 | [0.100, 1.187] | 0.091 | 0.342 | [0.099, 1.177] | 0.089 |
| 1 to 3 mo after x 2004 to 2007 | 1.291 | [0.349, 4.779] | 0.702 | 1.275 | [0.344, 4.722] | 0.716 |
| 4 to 6 mo after x 2004 to 2007 | 0.531 | [0.137, 2.054] | 0.359 | 0.521 | [0.135, 2.019] | 0.346 |
| 7+ mo after x 2004 to 2007 | 1.022 | [0.409, 2.552] | 0.964 | 1.013 | [0.406, 2.529] | 0.978 |
| 7 to 11 mo before x 2008 to 2015 | 1.107 | [0.352, 3.475] | 0.862 | 1.131 | [0.360, 3.551] | 0.834 |
| 4 to 6 mo before x 2008 to 2015 | 0.42 | [0.134, 1.314] | 0.136 | 0.423 | [0.135, 1.322] | 0.139 |
| 1 to 3 mo before x 2008 to 2015 | 0.413 | [0.147, 1.159] | 0.093 | 0.415 | [0.148, 1.162] | 0.094 |
| Month of mother’s death x 2008 to 2015 | 0.065 | [0.011, 0.382] | 0.002 | 0.066 | [0.011, 0.386] | 0.003 |
| 1 to 3 mo after x 2008 to 2015 | 1.000 | - | - | 1.000 | - | - |
| 4 to 6 mo after x 2008 to 2015 | 0.111 | [0.018, 0.698] | 0.019 | 0.112 | [0.018, 0.703] | 0.019 |
| 7+ mo after x 2008 to 2015 | 0.395 | [0.127, 1.224] | 0.108 | 0.396 | [0.128, 1.226] | 0.108 |
| Interactions between site and time period |  |  |  |  |  |  |
| AHDSS x 2004 to 2007 | 1.446 | [1.157, 1.808] | 0.001 | 1.449 | [1.159, 1.811] | 0.001 |
| AHDSS x 2008 to 2015 | 1.72 | [1.384, 2.138] | <0.001 | 1.721 | [1.385, 2.139] | <0.001 |
| Sex of child |  |  |  |  |  |  |
| Girl | 1.000 | - | - | 1.000 | - | - |
| Boy | 1.121 | [1.027, 1.223] | 0.01 | 1.121 | [1.027, 1.223] | 0.01 |
| Child age (months) |  |  |  |  |  |  |
| <1 | 1.000 | - | - | 1.000 | - | - |
| 1-6 | 0.364 | [0.311, 0.424] | <0.001 | 0.363 | [0.311, 0.424] | <0.001 |
| 7-23 | 0.175 | [0.151, 0.202] | <0.001 | 0.175 | [0.151, 0.202] | <0.001 |
| 24-59 | 0.035 | [0.029, 0.041] | <0.001 | 0.035 | [0.029, 0.041] | <0.001 |
| Multiple birth |  |  |  |  |  |  |
| Singleton | 1.000 | - | - | 1.000 | - | - |
| Multiple | 1.602 | [1.306, 1.964] | <0.001 | 1.595 | [1.301, 1.956] | <0.001 |
| Mother’s age at birth (years) |  |  |  |  |  |  |
| 15-19 | 1.000 | - | - | 1.000 | - | - |
| 20-24 | 1.064 | [0.935, 1.211] | 0.346 | 1.068 | [0.939, 1.216] | 0.317 |
| 25-29 | 1.455 | [1.259, 1.681] | <0.001 | 1.468 | [1.271, 1.696] | <0.001 |
| 30-34 | 1.31 | [1.097, 1.565] | 0.003 | 1.326 | [1.110, 1.583] | 0.002 |
| 35+ | 1.616 | [1.330, 1.962] | <0.001 | 1.641 | [1.351, 1.993] | <0.001 |
| Number of other children in household |  |  |  |  |  |  |
| 0 | 1.000 | - | - | 1.000 | - | - |
| 1 | 1.05 | [0.948, 1.164] | 0.347 | 1.051 | [0.949, 1.165] | 0.337 |
| 2 | 1.114 | [0.977, 1.272] | 0.108 | 1.119 | [0.981, 1.277] | 0.095 |
| 3+ | 1.295 | [1.111, 1.509] | 0.001 | 1.302 | [1.117, 1.517] | 0.001 |
| Father presence |  |  |  |  |  |  |
| Absent | 1.000 | - | - | 1.000 | - | - |
| Present | 0.479 | [0.424, 0.540] | <0.001 | 0.484 | [0.429, 0.546] | <0.001 |
| Older brother presence |  |  |  |  |  |  |
| Absent | 1.000 | - | - | 1.000 | - | - |
| Present | 0.918 | [0.820, 1.029] | 0.142 | 0.909 | [0.811, 1.018] | 0.098 |
| Older sister presence |  |  |  |  |  |  |
| Absent | 1.000 | - | - | 1.000 | - | - |
| Present | 0.911 | [0.813, 1.021] | 0.11 | 0.899 | [0.802, 1.008] | 0.068 |
| Maternal grandmother presence |  |  |  |  |  |  |
| Absent | 1.000 | - | - | 1.000 | - | - |
| Present | 0.776 | [0.680, 0.886] | <0.001 | 0.785 | [0.687, 0.896] | <0.001 |
| Maternal uncle presence |  |  |  |  |  |  |
| Absent | 1.000 | - | - | 1.000 | - | - |
| Present | 0.984 | [0.867, 1.117] | 0.804 | 0.992 | [0.874, 1.127] | 0.903 |
| Maternal aunt presence |  |  |  |  |  |  |
| Absent | 1.000 | - | - | 1.000 | - | - |
| Present | 0.953 | [0.839, 1.083] | 0.461 | 0.957 | [0.843, 1.088] | 0.504 |
| Household SES |  |  |  |  |  |  |
| Low |  |  |  | 1.000 | - | - |
| Middle |  |  |  | 0.929 | [0.836, 1.032] | 0.169 |
| High |  |  |  | 0.781 | [0.700, 0.871] | <0.001 |
|  | Parameter |  |  |  |  |  |
| $\sigma_{mother}^{2}$ | 1.122 | [0.883, 1.426] | - | 1.107 | [0.869, 1.410] | - |
