# Supplementary material for: Linking the timing of a mother’s and child’s death: Comparative evidence from two rural South African population-based surveillance studies, 2000–2015
Source: PLoS One. 2021 Feb 8;16(2):e0246671. doi: 10.1371/journal.pone.0246671 (PMC7869981; doi:10.1371/journal.pone.0246671)
Supplement: S2 Table — Causes of death categorised according to the South African burden of disease classification system. (DOCX) [file pone.0246671.s003.docx]

**S2 Table. Mother causes of death, classified by InterVA-5 based on VA.** Causes of death categorised according to the South African burden of disease classification system.

|  | N | (%) |
| --- | --- | --- |
| HIV/AIDS and TB | 2,280 | (59.2) |
| Other communicable | 327 | (8.5) |
| Noncommunicable | 535 | (13.9) |
| External | 77 | (2.0) |
| Unknown | 632 | (16.4) |
